# Dihydrotestosterone Enhances MICA-Mediated Immune Responses to Epstein–Barr Virus-Associated Gastric Carcinoma

**DOI:** 10.3390/cancers16183219

**Published:** 2024-09-21

**Authors:** Donghyun Seo, Hyeji Byun, Miyeon Cho, Sun Hee Lee, Sohyun Youn, Junho Lee, Inuk Jung, Hyosun Cho, Hyojeung Kang

**Affiliations:** 1 Vessel-Organ Interaction Research Center, Research Institute of Pharmaceutical Science, College of Pharmacy, Kyungpook National University, Daegu 41566, Republic of Korea; puppu04050@naver.com (D.S.); qkek0810@naver.com (H.B.); cmy1004g@knu.ac.kr (M.C.); ihappy278@knu.ac.kr (S.H.L.); 2 Department of Computer Science and Engineering, Kyungpook National University, Daegu 41566, Republic of Korea; yshggid@gmail.com (S.Y.); inukjung@knu.ac.kr (I.J.); 3 Department of Veterinary Surgery, College of Veterinary Medicine, Kyungpook National University, Daegu 41566, Republic of Korea; 2009045032@knu.ac.kr; 4 Duksung Innovative Drug Center, College of Pharmacy, Duksung Women’s University, Seoul 01369, Republic of Korea

**Keywords:** androgen receptor, dihydrotestosterone, Epstein–Barr virus, MICA, NF-κB

## Abstract

**Simple Summary:**

Epstein–Barr virus-associated gastric carcinoma (EBVaGC) is a type of stomach cancer linked to EBV infection. This study explores how the male hormone dihydrotestosterone (DHT) affects the immune response against EBVaGC. Using the SNU719 cell line, we treated the cells with DHT and observed an increased expression of the androgen receptor (AR) and the activation of the NF-κB pathway. This led to higher levels of MICA, a protein that interacts with immune cells like natural killer (NK) and T cells, enhancing their ability to kill EBVaGC cells. Importantly, this immune boost occurred without increasing harmful inflammatory signals. Our findings suggest that DHT enhances the immune system’s ability to target EBVaGC, providing a potential therapeutic approach by modulating androgen signaling to improve anti-tumor immunity.

**Abstract:**

Background: Epstein–Barr virus-associated gastric carcinoma (EBVaGC) is a subset of gastric cancers linked to EBV infection. While the role of male hormones in cancers such as prostate, breast, and ovarian cancers is well-studied, their impact on EBVaGC remains less understood. This study aims to examine the effect of dihydrotestosterone (DHT) on EBVaGC, particularly focusing on its influence on the immune response. Methods: The study utilized the SNU719 EBVaGC cell line. Cells were treated with DHT to assess androgen receptor (AR) expression and the activation of signaling pathways, including NF-κB. The expression of MHC class I polypeptide-related sequence A (MICA) and its interaction with the NKG2D receptor on NK and T cells was evaluated. Cytotoxicity assays were conducted to determine DHT’s effect on NK and T cell-mediated cytotoxicity, and proinflammatory cytokine gene expression was analyzed. Results: DHT significantly increased AR expression in EBVaGC cells and activated the NF-κB pathway, which led to increased transcription of target genes such as MICA and EBNA1. These changes enhanced the interaction with receptors on NK and T cells, thereby boosting their cytotoxicity against EBVaGC cells. Importantly, DHT did not upregulate proinflammatory cytokine genes. Conclusion: DHT enhances the immune response against EBVaGC by upregulating MICA and activating NK and T cells. These findings suggest potential therapeutic strategies targeting androgen signaling to improve anti-tumor immunity in EBVaGC.

## 1. Introduction

Gastric carcinoma is a significant global health concern, with various etiological factors contributing to its development. Among these, Epstein–Barr virus (EBV) infection has been identified as a key factor in a subset of gastric cancers, referred to as EBV-associated gastric carcinoma (EBVaGC) [1]. EBVaGC exhibits distinct molecular and clinical features, including unique immune evasion mechanisms and altered signaling pathways. Epstein–Barr virus is a ubiquitous herpesvirus that infects over 90% of the world’s population and establishes a lifelong latent infection within the host [2]. EBV is primarily transmitted through saliva and typically infects B lymphocytes and epithelial cells. Infected cells can harbor the virus in a latent state, during which the viral genome persists as an episome within the nucleus of the host cell, expressing a limited set of viral proteins that contribute to oncogenesis and immune evasion [3]. The latency-associated proteins, including EBV nuclear antigen 1 (EBNA1), latent membrane proteins (LMPs), and Epstein–Barr-encoded small RNAs (EBERs), are crucial for maintaining viral persistence and promoting cell proliferation [4].

A notable feature of EBVaGC is the expression of *EBNA1*, which is critical for the maintenance of the viral genome within infected cells. EBNA1 is known for its strong antigenicity, which can elicit immune responses, although the virus employs various strategies to evade immune detection [5]. Androgens, including dihydrotestosterone (DHT), play crucial roles in various physiological processes and have been implicated in the regulation of immune responses [6]. Androgen signaling occurs through the androgen receptor (AR), a type of nuclear receptor that, upon binding to androgens, translocates to the nucleus and influences gene expression [7]. The AR-DHT signaling pathway is pivotal in numerous biological processes, including the development and maintenance of male characteristics, the regulation of libido, and the modulation of muscle mass and bone density [8]. Additionally, AR-DHT signaling has been shown to influence cell proliferation, differentiation, and apoptosis, thereby playing a significant role in various cancers, including prostate [9] and gastric cancers.

Recent studies have suggested that DHT can modulate immune function by influencing signaling pathways within target cells, including the NF-κB pathway [10,11]. NF-κB is a key regulator of immune and inflammatory responses, controlling the expression of various genes involved in immune responses, cell proliferation, and survival [10]. This makes NF-κB a critical player in cancer progression and immune system interactions.

In this study, we investigate the effect of DHT on the expression of MHC class I-related chain A (*MICA*) in SNU719 cells, a human gastric cancer cell line derived from EBV-associated gastric carcinoma. MICA is a stress-induced ligand that is recognized by the NKG2D receptor on natural killer (NK) and CD8^+^ T cells [12]. NKG2D is an activating receptor that plays a crucial role in the immune system’s ability to detect and eliminate stressed, transformed, or infected cells [13]. The interaction between MICA and NKG2D triggers the cytotoxic activity of these immune cells, enhancing their ability to target and destroy tumor cells.

Our findings demonstrate that treatment with DHT leads to the upregulation of NF-κB, mediated by androgen receptor signaling, which in turn increases the expression of *MICA* in SNU719 cells. This increase in MICA expression enhances the ability of NK and CD8+ T cells to identify and attack SNU719 cells, making the tumor cells more vulnerable to immune-mediated cytotoxicity.

Understanding the mechanisms by which DHT influences *MICA* expression and the subsequent immune response in the context of EBVaGC can provide valuable insights into potential therapeutic strategies for enhancing anti-tumor immunity. This study aims to elucidate the role of DHT in modulating tumor cell susceptibility to immune-mediated cytotoxicity, particularly through the AR and NF-κB pathways, and to explore the potential implications for cancer treatment in EBVaGC. Moreover, we consider the role of EBNA1 antigenicity in shaping the immune landscape of EBVaGC, further underscoring the importance of immune modulation in treatment approaches.

## 2. Materials and Methods

### 2.1. Cells and Reagents

SNU719 and YCCEL1 gastric carcinoma cell lines (EBVaGC) were maintained in RPMI 1640 medium (HyClone, GE Healthcare, Pittsburgh, PA, USA) supplemented with 10% fetal bovine serum (HyClone), 1% antibiotic–antimycotic solution (Gibco, Thermo Fisher Scientific, Waltham, MA, USA), and 1% GlutaMAX (Gibco) for enhanced growth and viability. HEK293T cells were cultured in DME medium (HyClone) supplemented with 10% fetal bovine serum (HyClone), 1% antibiotics/antimycotics (Gibco), and 1% GlutaMAX (Gibco). NK-92 cells were obtained as a gift from Dr. Cho at Duksung Women’s University and cultured in alpha minimum essential medium (Gibco) supplemented with rIL-2 (100–200 U/mL, 589102, BioLegend, San Diego, CA, USA), 0.19 mM 2-mercaptoethanol, 10% fetal bovine serum (HyClone), and 1% penicillin–streptomycin (Gibco). Peripheral blood mononuclear cells (PBMCs) were isolated from whole blood obtained from healthy donors by Ficoll–Paque density gradient centrifugation and used to generate anti-CD3 antibody (Ab, OKT3, 317326, BioLegend)-activated PBMCs and EBV-specific PBMCs. OKT3-activated PBMCs were generated from PBMCs (1 × 10^6^ cells/mL) using OKT3 (100 ng/mL) and cultured in a humidified incubator at 37 °C under 5% CO_2_ in RPMI-1640 medium supplemented with 10% heat-inactivated human AB serum (Sigma-Aldrich, Burlington, MA, USA), 1% penicillin–streptomycin, IL-2 (20 IU/mL), IL-7 (20 ng/mL, 207-IL-010, R&D Systems), and IL-15 (20 ng/mL, 247-ILB-005, R&D Systems) for seven days. The study protocol was approved by Kyungpook National University (Daegu, Republic of Korea), and the blood donors provided written informed consent (permit number, 2022-02-036). All cells were cultured at 37 °C under 5% CO_2_ and 95% humidity.

### 2.2. ChIP-qPCR Assay

The ChIP-seq assay was performed against the NF-κB p65 (p65) protein using SNU719 cells (3 × 10^7^ cells/sample) treated with 100 nM DHT for 0, 0.5, and 1 h, followed by formaldehyde crosslinking. The resultant SNU719 cell lysates were sonicated to obtain DNA fragments of approximately 100–500 bp length. Immunoprecipitation was performed using 2.5 μg of either rabbit anti-p65 antibody (Ab) (Cell Signaling Technology, Danvers, MA, USA) or control rabbit IgG Ab (GeneTex, Irvine, CA, USA), followed by overnight incubation with Ab-coated Dynabeads protein A/G (Invitrogen, Carlsbad, CA, USA). The incubated beads were washed five times with ChIP-seq wash buffer (50 mM HEPES [pH 7.5], 500 mM LiCl, 1 mM EDTA, 1% NP-40, 0.7% Na deoxycholate, and 1× protease inhibitors) and then washed once with 50 mM NaCl in TE buffer. Immunoprecipitated DNA was eluted using ChIP-seq elution buffer (50 mM Tris–HCl [pH 8], 10 mM EDTA, and 1% sodium dodecyl sulfate [SDS]), reverse-crosslinked at 65 °C, treated with RNase A (0.2 mg/mL) and proteinase K (0.2 mg/mL), purified with phenol and chloroform, and subjected to quantitative polymerase chain reaction (qPCR) validation using the ChIP assay. The ChIP samples were used to determine the p65 enrichment through a qPCR assay. The p65 enrichment was investigated for the *AR* exon 1 region (chrX: 67,545,300~67,545,350 bp) and *MICA* promoter region (Chr6: 31,394,900~31,385,050 bp), respectively. Information on the primer sequences used in the ChIP assay was to be submitted upon request.

### 2.3. ChIP-Seq Assay

Among the above p65 ChIP samples, the sample treated with 100 nM DHT for 30 min showed the strong p65 enrichment on the SNU719 genome. Therefore, this 30 min-treated p65 ChIP sample was analyzed by a next-generation sequencing (NGS) assay to identify the genome-wide locus where the p65 protein is enriched. As a control, IgG ChIP samples were also analyzed by NGS assay to identify non-selective enrichment. The validated ChIP samples were further isolated by agarose gel purification, ligated to primers, and subjected to Illumina-based sequencing using the manufacturer’s protocol (Illumina, San Diego, CA, USA) for ChIP-seq analysis. ChIP-seq reads were mapped to the EBV wild-type reference genome (NC 007605) using Bowtie software (version 2.3.4.1). The MAC2 tool was used for peak calling [12].

### 2.4. AR Depletion by CRISPR/Cas9 Genome Editing

The CRISPR/Cas9 system was used to generate SNU719 cells with the depletion of *AR*. The sgRNA sequence was designed using the web tool of the Centre for Organismal Studies Heidelberg (COS): CCTop (https://crispr.cos.uni-heidelberg.de/) accessed on 1 February 2021. The following sgRNA sequence was utilized to target the AR gene: 5′-CACCgTGCACTTCCATCCTTGAGCTCA-3′. The sgRNA targeting human AR (NC_000023.11) was designed and inserted into the LentiCRISPRv2 vector (catalog number 52961, Addgene, Watertown, MA, USA). HEK293T cells were then cotransfected with the LentiCRISPRv2 or LentiCRISPRv2-AR sgRNA plasmid, along with lentiviral packaging plasmids (psPAX2 and pMD2.G), using TurboFect Transfection Reagent (R0531, Thermo Fisher Scientific), and incubated for 72 h.

The viral supernatants were collected, filtered, and used to infect SNU719 cells for 24 h. Afterward, the supernatants were replaced with fresh RPMI medium, and the cells were treated with 0.5 μg/mL puromycin every 2 to 3 days. Knockout efficiency was validated by analyzing protein expression. The resulting SNU719 cells with AR depletion were designated as SNU719_AR(−) cells (Supplementary Figure S1A,B). SNU719 cells transfected with a lentivirus containing only the LentiCRISPRv2 vector were named SNU719_AR(+) cells.

### 2.5. Depletion of MICA and HLA-A Transcripts Using the shRNA Gene Knockdown System

The shRNA lentiviral system was used to create SNU719 cells with the depletion of the MHC class I chain-related protein A (*MICA*) transcript or *HLA-A* transcript. The shRNA sequence was designed through a web tool based on the shRNA design algorithm (https://portals.broadinstitute.org/gpp/public/seq/search) accessed on 1 August 2021. The sequence used to knockdown *MICA* was 5′-GGCCATGAACGTCAGGAATTT-3′, and the sequence used to knockdown *HLA-A* was 5′-CTCCCACTCCATGAGGTATTT-3′. An shRNA targeting human *MICA* and an shRNA targeting human *HLA-A* were designed and cloned into the pLKO.1 vector (catalog number 8453, Addgene). The resultant pLKO.1 vectors were named pLKO.1-MICA shRNA and pLKO.1-HLA-A shRNA vectors, respectively. The pLKO.1 vector, pLKO.1-MICA shRNA vector, and pLKO.1-HLA-A shRNA vector were cotransfected with lentiviral packaging plasmids (psPAX2 and pMD2.G) into HEK293T cells for 36 h using Lipofectamine 2000 transfection reagent (11668019, Invitrogen). The viral supernatant was harvested, filtered, and then used to infect SNU719 cells for 24 h at an MOI of 1. The viral supernatant was then replaced with fresh RPMI medium, and infected cells were selected by treatment with 1.5 µg/mL puromycin every 2 to 3 days.

The knockdown efficiency of MICA was assessed using reverse-transcription qPCR (RT-qPCR) and Western blot analysis, which confirmed significantly reduced levels of mRNA and protein expression, respectively (Supplementary Figure S1C,D). Similarly, the knockdown efficiency of HLA-A was confirmed using the same methods, showing decreased HLA-A expression (Supplementary Figure S1E,F).

The resultant SNU719 cells with the depletion of *MICA* and *HLA-A* transcripts were named SNU719_MICA(−) and SNU719_HLA-A(−) cells, respectively. SNU719 cells infected with a lentivirus and packaged only with the pLKO.1 vector as a control group were named SNU719_pLKO.1 cells.

### 2.6. DHT Treatment

DHT (#A8380, Sigma-Aldrich) was dissolved in ethanol and treated at various concentrations, such as 100 nM and 5 μM. Ethanol was treated as a negative control at a concentration of 0.1%. Phenol red-free RPMI-1640 medium (Gibco) supplemented with 5% charcoal-stripped FBS (CS-FBS, Gibco), 1% antibiotic–antimycotic, and 1% GlutaMAX was named androgen-free medium and used for in vitro DHT treatment. EBVaGC cell lines were transferred to androgen-free medium after attachment and stabilized for 72 h. After 72 h, the cells were plated in an appropriate quantity according to the experiment and cultured in fresh androgen-free medium for 24 h with either DHT or ethanol (EtOH) treatment. Immune cells, such as PBMCs and NK-92 cells, were plated without prior stabilization in an appropriate quantity according to the experiment and cultured in androgen-free medium for 24 h with either DHT or EtOH.

### 2.7. Lactate Dehydrogenase (LDH) Release-Based Cytotoxicity Test

The cytotoxicities of effector cells, such as NK-92 cells and EBV peptide-stimulated PBMCs (EBNA1 PBMCs and LMP2A PBMCs), against target cells, such as SNU719 and YECCL1 cells, were analyzed using the CyQUANT™ LDH Cytotoxicity Assay Kit (#C20300, Invitrogen), according to the manufacturer’s protocol. The target cells were plated at 5 × 10^3^ cells/well in a 96-well plate and incubated overnight at 37 °C under humidity. The next day, effector cells were added at various ratios to the tumor cells (1:0, 1:1, 1:5, and 1:15 target cell/effector cell ratio [T:E]). After 4 or 24 h of coculture, the mixture was centrifuged at 250× *g* for 5 min at room temperature. Then, a 50 μL aliquot of the medium was used for the LDH released-based cytotoxicity assay using the LDH Cytotoxicity Assay Kit (Invitrogen). Experimental LDH release was corrected by subtracting the LDH amount spontaneously released in the cells at the corresponding dilutions. The percentage of cytotoxicity was calculated as follows: % Cytotoxicity = (Experimental value − Effector cell spontaneous control − Target cell spontaneous control)/(Target cell maximum control − Target cell spontaneous control) × 100. The coculture of SNU719 target cells and NK-92 effector cells for 24 h induced up to 15% cytotoxicity depending on T:E. The cytotoxicity induced at each T:E could be partially observed using an optical microscope.

### 2.8. Western Blot Analysis

Western blot assay was performed to identify molecular events related to immune response and signal transduction in DHT-treated SNU719 cells. SNU719 cells were treated with DHT at various concentrations for 48 h, and total protein was harvested from treated cells as described in the previous protocol. Briefly, 5 × 10^6^ cells were lysed with 200 μL radioimmunoprecipitation assay (RIPA) lysis buffer supplemented with protease inhibitors (PI, Sigma) and phenylmethylsulfonyl fluoride (PMSF, Sigma) and further fractionated using a Biolutor sonicator (Cosmobio, Tokyo, Japan) set to 30 s on/off pulses for 5 min. Proteins in cell lysates were measured by BCA assay: proteins were separated by 10% SDS-polyacrylamide gel electrophoresis and transferred to 0.45 μm polyvinylidene fluoride membranes (Millipore). The membrane was probed with antibodies (Abs) against cellular proteins. For the primary detection of target proteins on the membrane, anti-AR Ab (Cell Signaling Technology), anti-p-p65 Ab (Cell Signaling Technology), anti-p65 Ab (Cell Signaling Technology), anti-lamin B1 Ab (Cell Signaling Technology), anti-MICA/B Ab (Cell Signaling Technology) and anti-beta-actin Ab (Santa Cruz Biotechnology, Dallas, TX, USA) were used.

To detect the primary antibodies, goat anti-mouse IgG-HRP and goat anti-rabbit IgG-HRP (both from Genetex) were employed as secondary antibodies. The antibody-bound proteins were visualized using an enhanced chemiluminescence (ECL) detection reagent (Bio-Rad, Hercules, CA, USA). Each Western blot analysis was performed in triplicate, and representative results of this analysis were analyzed using ImageJ 1.54g.

### 2.9. RT-qPCR Assay

To confirm the knockdown efficiencies of *AR* and *MICA* and the effects of DHT on the SNU719 cell transcriptome, real-time RT-qPCR assays were performed.

Total RNA was extracted from the cells using the RNeasy Mini Kit (Qiagen, Germantown, MD, USA). The isolated RNA was then reverse transcribed into cDNA with SuperScript III Reverse Transcriptase (Invitrogen).

The resulting cDNA was diluted in nuclease-free water and utilized for analyzing cellular gene expression via real-time qPCR (LightCycler 96, Roche, Basel, Switzerland). Actin mRNA levels were measured in each sample as an internal control. To ensure accuracy, RT-qPCR was also conducted on RNA samples without reverse transcription, serving as a negative control for each reaction.

The gene primer set for real-time qPCR was as follows: *MICA*, forward: CGG GGA TCC ATG GAA GTG CAG TTA GGG CT and reverse: CCG CTC GAG TCA CTG GGT GTG GAA ATA GA; *AR*, forward: GGA TGG AAG TGC AGT TAG GG and reverse: GAG GTG CTG CGC TCG CGG. Upon request, we can provide information about the primer sets used in RT-qPCR to detect transcripts of other cellular and EBV genes.

### 2.10. Generation of EBV-Specific PBMCs

PBMCs were obtained from donors (Kyungpook National University Chilgok Hospital IRB# 2022-02-036 IRB# 2022-02-036). PBMCs were stimulated with peptide pools consisting of 15-mers with 11 overlapping amino acids. The EBV EBNA-1 peptide pool (#130-093-613, Miltenyi Biotec, Bergisch Gladbach, Germany) was used to generate EBNA-1-specific PBMCs (named EBNA1 PBMCs), while the EBV LMP2A peptide pool (130-093-615, Miltenyi Biotec) was used to generate LMP2A-specific PBMCs (named LMP2A PBMCs). The lyophilized peptide pools were dissolved in ultrapure water (UPW) and kept at −80 °C for storage. To generate these EBV-specific PBMCs, PBMCs (1 × 10^6^ cells/mL) were cultured in a humidified incubator at 37 °C with 5% CO_2_ in RPMI-1640 medium supplemented with 10% heat-inactivated human AB serum, 1% penicillin–streptomycin, IL-2 (20 IU/mL), IL-4 (1000 IU/mL, 204-IL-010, Miltenyi Biotec), IL-7 (20 ng/mL), IL-15 (20 ng/mL), granulocyte macrophage colony-stimulating factor (GM-CSF, 1000 IU/mL, 215-GM-010, Miltenyi Biotec), and each peptide pool (5 ng/peptide/mL) for 14–17 days.

### 2.11. Co-Immunoprecipitation (Co-IP) Assay

Co-IP and Western blot assay was performed according to a previously described protocol [13]. To analyze the interaction between NF-κB subunits and AR proteins, SNU719 cells were treated with 100 nM DHT for 48 h. The cells were lysed in RIPA lysis buffer (25 mM Tris–HCl [pH 7.6], 150 mM NaCl, 2% Nonidet P-40, 1% sodium deoxycholate, 10% SDS, and protease inhibitors) on ice for 30 min, and the cell debris was cleared by centrifugation at 10,000× *g* at 4 °C. The resulting supernatants were co-immunoprecipitated with anti-p65 Ab (Cell Signaling Technology) or anti-NF-κB2 p100/52 Ab (Cell Signaling Technology) at 4 °C overnight; rabbit IgG was used as a negative control. The immunocomplexes were collected using Dynabeads Protein G (Invitrogen) at 4 °C overnight and washed thrice with ice-cold lysis buffer. The immunoprecipitates were eluted with Laemmli sample buffer, and the samples were analyzed by Western blot analysis.

### 2.12. NF-κB Activity Assay

To analyze the promoter binding activity of NF-κB, SNU719 cells were transfected with the pSI-Check2-hRluc-NFκB-firefly reporter plasmid (catalog number 106979, Addgene) using Lipofectamine. Forty-eight hours post-transfection, the SNU719 cells were exposed to DHT for either an additional 6 or 24 h.

Luciferase activity, indicative of NF-κB binding activity, was measured using the Dual-Luciferase Reporter Assay System (Promega, Madison, WI, USA) according to the manufacturer’s instructions. Relative luciferase activity was calculated by normalizing firefly luciferase activity to renilla luciferase activity.

### 2.13. Cytokine Array Assay

PBMCs, SNU719 cells and NK-92 cells were treated 100 nM DHT for 24 h. Mediums were collected, and cell debris was removed by centrifugation at 3000 rpm for 5 min. The supernatant was analyzed with a Proteome Profiler Human Cytokine Array kit (R&D Systems, Minneapolis, MN, USA) according to the manufacturer’s instructions.

### 2.14. Immunofluorescence Assay (IFA)

For the immunofluorescence assay (IFA), SNU719 cells were cultured on coverslips in 24-well plates and treated with 1000 nM DHT for 48 h. Cells were fixed with 4% paraformaldehyde for 20 min and permeabilized with 0.25% Triton X-100 in PBS for 15 min. Blocking was performed with 1% BSA in PBS containing 0.1% Tween 20 for 30 min. The samples were then incubated with a MICA antibody (1:40) overnight at 4 °C. After washing the coverslips three times in PBS, they were treated with Alexa-488 (Thermo Fisher Scientific) for 1 h at room temperature to detect MICA. Following three washes with PBT (PBS containing 0.5% Triton X-100), coverslips were mounted using DAPI (SouthernBiotech, Birmingham, AL, USA) and analyzed via immunofluorescence confocal microscopy.

### 2.15. FACs Analysis

To observe the expression of *HLA-A*2 and *EBNA1* upon DHT treatment in SNU719, we performed FACS analysis. SNU719 cells treated with 500 nM and 5 uM of DHT for 48 h were trypsinized to isolate single cells and washed with FACS buffer (Dulbecco’s phosphate-buffered saline [DPBS] containing 0.1% bovine serum antigen [BSA], 2 mM ethylene-diamine-tetraacetic acid [EDTA]). Next, cells were blocked with 1% BSA for 10 min at room temperature (to prevent non-specific binding). Cells were then treated with 10 μg/mL FITC mouse anti-human HLA-A2 antibody (clone BB7.2, BD Biosciences, Franklin Lakes, NJ, USA, #551285), 1 μg/mL EBV-EBNA-1 antibody (clone 1EB12, Santa Cruz Biotechnology, #sc-81581 AF647) and 10 μg/mL MICA antibody (clone 2C10, Santa Cruz Biotechnology, #sc-23870 PE) for 30 min at 4 °C. Cell analysis was performed using a MACS Quant Analyzer 10 (Milteny Biotec, Bergisch Gladbach, Germany), with unstained cells used for negative controls.

### 2.16. Statistical Analysis

All experiments were performed at least three times to ensure the reproducibility of results. Statistical analyses were conducted using the Prism software package (GraphPad Prism 10.3.0, San Diego, CA, USA). Two-tailed Student’s *t*-tests (Microsoft, Redmond, WA, USA) were employed to assess differences between two groups, while one-way analysis of variance (ANOVA) followed by Tukey’s multiple comparison test was used for comparisons among three or more groups. Statistically significant differences identified by the two-tailed Student’s *t*-test are indicated by asterisks (*), and significant differences from one-way ANOVA and Tukey’s test are represented by different uppercase or lowercase letters.

## 3. Results

### 3.1. DHT Regulates NF-κB Activity

The androgen receptor (AR) and NF-κB have complex interactions, which have been explored in various contexts in multiple studies. Key interactions include mutual inhibition, where AR activation can suppress NF-κB transcriptional activity and vice versa [14,15]. They also co-regulate certain target genes, often interacting in the regulation of genes related to inflammation, cell growth, and survival, with their effects varying depending on the cellular environment and specific stimuli [16]. In the tumor microenvironment, particularly in cancers such as prostate cancer, AR signaling can inhibit NF-κB activity to exert anti-inflammatory effects, while NF-κB activation can suppress AR signaling, promoting cell survival and growth [17,18]. The interaction between AR and NF-κB can differ across cell types and pathological conditions. Some studies show that in certain cancer cells, AR may regulate cell survival and inflammatory responses through NF-κB activation [19]. These interactions are complex and dependent on various signaling pathways and intracellular environments, making them critical targets for disease treatment.

**Figure 1:**
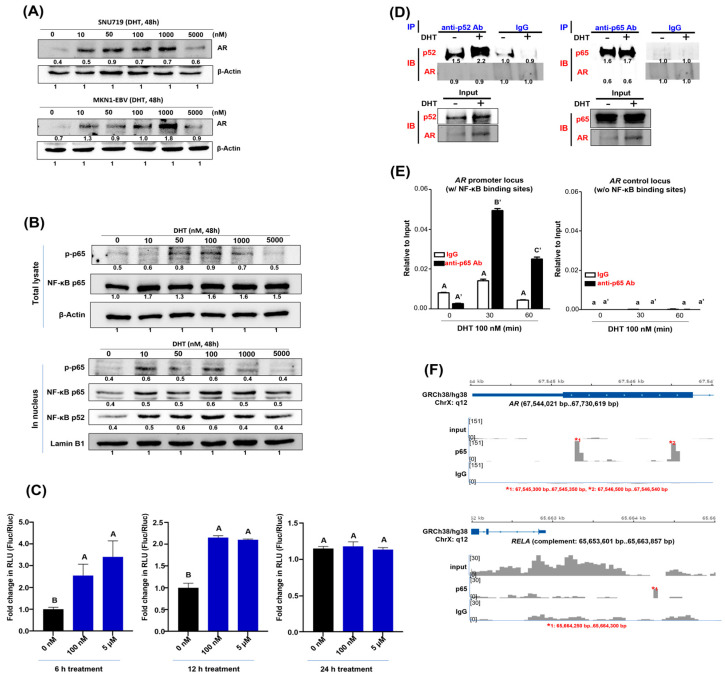
**DHT regulates NF-κB activity.** (**A**) *AR* upregulation in SNU719 (**upper panel**) and MKN1-EBV (**bottom panel**) cells treated with DHT for 48 h. (**B**) Expression of the NF-κB p65 (p65) and NF-κB p52 (p52) proteins in the total lysate and the nucleus of SNU719 cells treated with 100 nM DHT for 48 h. Expression changes of phosphorylated p65 (p-p65) and p65 proteins in the total lysate were further investigated; p-p65 protein was p65 phosphorylated at serine 536 subunit. In addition, expression changes of p52 protein in the nucleus were additionally investigated. (**C**) Analysis of changes in NF-κB activity in DHT-treated SNU719 cells. The pSI-Check2-hRluc-NFκB-firefly reporter plasmid was transfected into SNU719 cells for 48 h, then followed by 6 h (**left**), 12 h (**middle**) and 24 h (**right**) of DHT treatment, and finally luciferase activity was analyzed. (**D**) To analyze the interaction between NF-κB subunit (p52, p65) and AR proteins, a co-immunoprecipitation (co-IP)-Western blot assay was performed. First, IP was performed with anti-p52 Ab and Western blot assay was performed with anti-AR Ab (**left**). Next, IP was performed with anti-p65 Ab, and Western blot assay was performed with anti-AR Ab (**right**). (**E**) ChIP assay showing binding of the p65 protein to the *AR* promoter locus (5′ promoter site of *AR* exon 1) and *AR* control locus (3′ intron site of *AR* exon 1) in SNU719 cells. SNU719 cells were treated with 100 nM DHT for 0, 30, and 60 min and then subjected to p65 ChIP-PCR assay. The p65 enrichment was examined at the *AR* promoter locus, known to contain NF-κB binding sites, and the *AR* control locus, known not to contain NF-κB nonbinding sites. ChIP assay results for the *AR* promoter locus, a known NF-κB binding site, are shown in the figure. Groupings of IgG ChIP samples are labeled with capitalized letters (A), while groupings of anti-p65 antibody ChIP samples are labeled with capitalized letters (A′). For the *AR* control locus, which is a non-binding site for NF-κB, IgG ChIP sample groupings are indicated by lowercase letters (a), and anti-p65 antibody ChIP sample groupings are indicated by lowercase letters (A′). (**F**) The p65 enrichment for upregulation of *AR* and *RELA*. p65 ChIP-seq analysis was performed to evaluate the p65 enrichment sites in the genome of SNU719 cells treated with 10b0 nM DHT for 30 min. The reference human genome used was GRCH38/fg38. The p65 enrichment was assessed in the *AR* promoter locus located on chromosome X, q12 (**top**) and near the *RELA* promoter locus located on chromosome 11, q13.1 (**bottom**). We performed a one-way ANOVA test for comparisons involving more than three groups, followed by Tukey’s multiple comparison test if the results were significant. The results were presented using a compact letter display, with both uppercase and lowercase letters used to denote significant differences. In the context of previous studies, we also wondered how DHT-AR signaling affects NF-κB expression in EBVaGC cells, which we investigated as follows. First, we investigated how DHT regulates AR protein expression in SNU719 cells by Western blot assay. AR protein expression in SNU719 cells treated with DHT for 48 h showed a nearly concentration-dependent increase (Figure 1A).

Second, we evaluated the levels of NF-κB p65 and its phosphorylated form (phospho-p65) in SNU719 cells following 48 h DHT treatment at varying concentrations. The expression of phospho-p65 and p65 proteins increased in the total lysate on treatment with 50–1000 nM DHT (Figure 1B). Similarly, we assessed the expression of phospho-p65, p65 and NF-κB p52 (p52) proteins in the nucleus of SNU719 cells treated with different concentrations of DHT for 48 h. The expression of phospho-p65, p65 and p52 proteins increased in the nucleus on treatment with 10–1000 nM DHT (Figure 1B). These results imply that DHT-mediated *AR* overexpression is related to DHT-mediated NF-κB overexpression.

Third, we investigated whether DHT also enhances the activity of NF-κB. SNU719 cells transfected with the pSI-Check2-hRluc-NFκB-firefly reporter plasmid were treated with DHT for 6 or 24 h, and the activity of NF-κB was measured by luciferase assay. The results showed that NF-κB activity was statistically significantly increased in SNU719 cells treated with DHT for 6 h and 12 h, while NF-κB activity was unchanged in SNU719 cells treated with DHT for 24 h (Figure 1C).

Fourth, a previous study reported that AR could physically interact with NF-κB in cells by co-immunoprecipitation (CO-IP) assay and that AR and NF-κB can occupy the same DNA regulatory regions by ChIP assay [8]. This previous study led us to analyze whether DHT contributes to the interaction of AR with NF-κB in EBVaGC cells by CO-IP and Western blot assays. We performed a Co-IP assay with anti-p52 Ab and Western blot assay with anti-AR Ab using SNU719 cells treated with 100 nM DHT for 48 h. We did not detect any interaction between AR and p52 proteins (Figure 1D). Moreover, we performed a Co-IP assay with anti-p65 Ab and Western blot assay with anti-AR Ab using SNU719 cells treated with 100 nM DHT for 48 h. We did not detect any interaction between AR and p65 proteins (Figure 1D). These results suggest that DHT increases the expression of AR and NF-κB proteins but does not contribute to the interaction of AR with NF-κB. Therefore, DHT-mediated AR signaling is indirectly related to the transcriptional activity of NF-κB.

Fifth, we wanted to further analyze whether AR and NF-κB upregulated by DHT have a regulatory interaction with each other. O’Callaghan et al. performed EMSA and showed that 1 h treatment with TNF-α increased NF-κB binding to the *MICA* promoter, and that NF-κB binding to the *MICA* promoter upregulated *MICA* [20]. Based on these experiments, we also investigated whether the p65 protein binds to the *AR* promoter locus after 1 h of DHT treatment by ChIP-qPCR assay (Figure 1E). The p65 protein was statistically significantly enriched at the *AR* promoter locus in SNU719_AR(+) cells treated with 100 nM DHT for 30 min. While there was also significant enrichment in the 60 min treatment group, the p65 protein was enriched much more strongly in the 30 min treatment group. These results suggest that DHT increases NF-κB binding at promoter sites, which is essential as a transcriptional activator in SNU719 cells.

Sixth, after finding by p65 ChIP-assay that the p65 protein is enriched at the *AR* promoter locus via DHT-AR signaling, we analyzed the p65 ChIP products on a genome-wide scale by NGS assay, called p65 ChIP-seq assay. Specifically, p65 ChIP products harvested from SNU719_AR(+) cells treated with DHT for 30 min were used for an NGS assay. We found 27,410 significant enrichment peaks in the input sample, 2829 significant enrichment peaks in the anti-p65 ChIP sample, and 1655 significant enrichment peaks in the IgG chip sample. We also analyzed the p65 enrichment in several important gene regions. We found that the p65 protein selectively binds to the *AR* promoter locus (5′ promoter site of *AR* exon 1, Figure 1F top) and the *RELA* promoter locus (5′ promoter site of *RELA* exon 1, Figure 1F bottom). The p65 protein was almost 10-fold more strongly enriched in the *AR* promoter locus (putative promoter) than in its own *RELA* promoter locus. Based on the above results, it is suggested that DHT upregulates NF-κB and that increased NF-κB binds to the *AR* promoter and increases *AR* transcription.

### 3.2. DHT-Induced NF-κB Upregulates MICA

**Figure 2:**
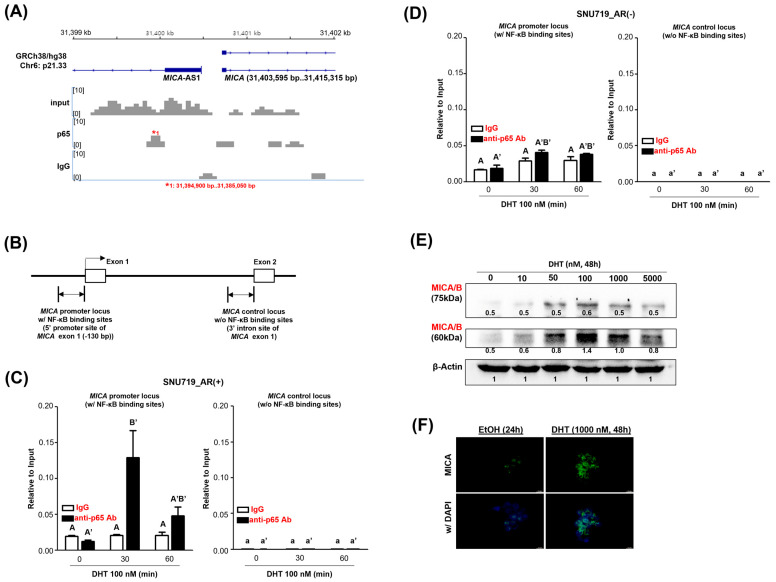
**DHT-induced NF-κB regulates MICA.** (**A**) The p65 enrichment for the MICA upregulation. To investigate the p65 enrichment sites in the genome of SNU719 cells treated with 100 nM DHT for 30 min, we performed p65 ChIP-seq analysis. We examined the p65 binding pattern enriching the promoter of MICA located at p21.33 on chromosome 6. (**B**) Genomic structure of the MICA promoter locus (the 5′ promoter site of MICA exon 1) expected to be bound by p65 protein and the MICA control locus (the 3′ intron site of MICA exon 1) not expected to be bound by p65 protein. (**C**) Re-validation by ChIP-qPCR assay of p65 binding to the MICA promoter locus and the MICA control locus in SNU719_AR(+) cells. SNU719_AR(+) cells were treated with 100 nM DHT for 0, 30, and 60 min and then subjected to p65 ChIP-PCR assay. The p65 enrichment was examined at the MICA promoter locus, known to contain NF-κB binding sites, and the MICA control locus, known not to contain NF-κB nonbinding sites. ChIP assay results for the *MICA* promoter locus, a known NF-κB binding site, are shown in the figure. Groupings of IgG ChIP samples are labeled with capitalized letters (A), while groupings of anti-p65 antibody ChIP samples are labeled with capitalized letters (A′). For the *MICA* control locus, which is a non-binding site for NF-κB, IgG ChIP sample groupings are indicated by lowercase letters (a), and anti-p65 antibody ChIP sample groupings are indicated by lowercase letters (A′). (**D**) Re-validation by ChIP-qPCR assay that p65 protein binds to the MICA promoter locus and the MICA control locus in SNU719_AR(−) cells. SNU719_AR(−) cells were treated with 100 nM DHT for 0, 30, and 60 min and then subjected to p65 ChIP-PCR assay. The p65 enrichment was examined at the MICA promoter locus and the MICA control locus. (**E**) Analysis of changes in the expression of 75-kDa and 65-kDa MICA/B proteins in SNU719 cells treated with different concentrations of DHT. (**F**) IFA assay detecting the increase in MICA expression induced by 1000 nM DHT treatment. We performed a one-way ANOVA test for comparisons involving more than three groups, followed by Tukey’s multiple comparison test if the results were significant. The results were presented using a compact letter display, with both uppercase and lowercase letters used to denote significant differences.

The p65 ChIP-seq assay described previously showed that the p65 protein binds to the promoters of various genes. As one of these, the p65 protein was found to be enriched at the MICA promoter locus (Figure 2A). The binding of the p65 protein to the *MICA* promoter locus was approximately three times weaker than the binding to its own *RELA* promoter locus.

As the binding of the p65 protein to the *MICA* promoter locus was rather weak, we performed another p65 ChIP-qPCR assay under the same conditions. The p65 ChIP-qPCR assay examined the binding of the p65 protein to the *MICA* promoter locus (5′ promoter site of *MICA* exon 1 (a 130 bp upstream region of *MICA* exon 1 region) and the MICA control locus (3′ intro site of *MICA* exon 1, an intronic region adjacent to the 5′ end of exon 2) (Figure 2B). In SNU719_AR(+) cells treated with 100 nM DHT for 30 min, the p65 protein bound statistically significantly to the *MICA* promoter locus, and in SNU719_AR(+) cells treated for 60 min, the p65 protein bound statistically non-significantly to the *MICA* promoter locus (Figure 2C). However, at the *MICA* control locus, the p65 protein barely bound.

Like SNU719_AR(+) cells, SNU719_AR(−) cells were treated with 100 nM DHT for 30 and 60 min, respectively, to investigate whether the p65 protein enriches at the *MICA* promoter locus by p65 ChIP-qPCR assay (Figure 2D). As expected, in DHT-treated SNU719_AR(−) cells, the p65 enrichment at the *MICA* promoter locus was similar to that of the control, and the p65 enrichment was almost absent at the *MICA* control locus.

Since NF-κB acts primarily as a transcriptional activator [21], we hypothesized that the p65 enrichment to the *MICA* promoter locus upregulates *MICA,* and we tested this hypothesis by Western blot assay. MICA/B expression levels were analyzed in SNU719 cells after 48 h of treatment with DHT in a range of concentrations. MICA/B was strongly expressed in SNU719 cells treated with 50, 100, and 1000 nM DHT (Figure 2E).

Next, we re-validated the DHT-mediated increase in the expression of MICA protein by immunofluorescence assay (IFA). To clearly observe MICA protein expression in SNU719 cells, we treated them with 1000 nM DHT for 48 h. The results showed that MICA protein was expressed much stronger in DHT-treated SNU719 cells compared to the control (Figure 2F). The above results suggested that *MICA* was upregulated by DHT, which acted as a positive regulator by attaching NF-κB to the *MICA* promoter and enhancing the expression of MICA protein.

### 3.3. DHT Increases the Cytotoxicity of NK Cells

In the above experiments, DHT increased NF-κB expression, increased the p65 enrichment to the *MICA* promoter site, and ultimately increased the expression of MICA protein. MICA is a ligand for NKG2D, a NK-cell-activating receptor. The p65 ChIP-seq assay above showed that the p65 protein enriched at the promoter of RAET1G, another ligand of NKG2D, and *RAET1G* transcription was also predicted to be increased (Supplementary Figure S2).

**Figure 3:**
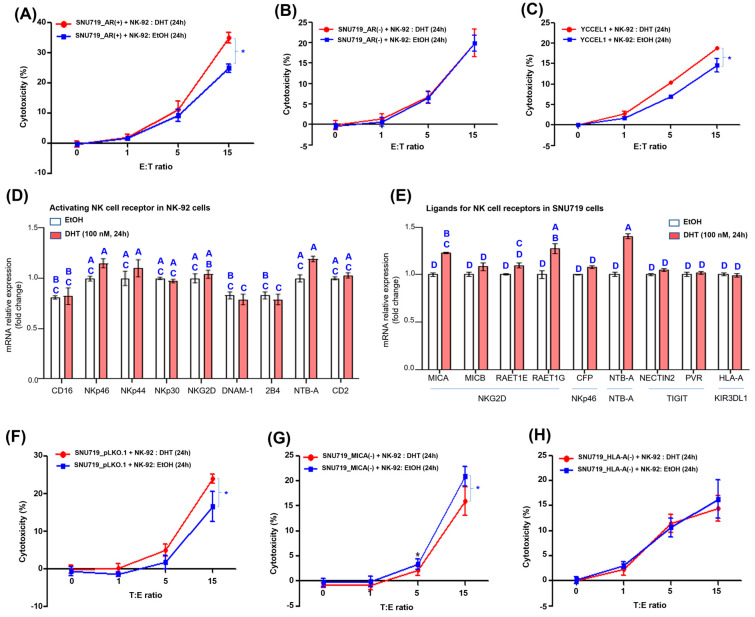
**DHT increases the cytotoxicity of NK cells.** (**A**) Investigation of cytotoxicity of NK-92 cells against SNU719_AR(+) cells treated with 100 nM DHT for 24 h by LDH release-based cytotoxicity assay. Cytotoxicity of NK-92 cells against SNU719_AR(+) cells treated with EtOH for 24 h was used as a control. Statistically significant differences identified by the two-tailed Student’s *t*-test are indicated by asterisks (*). (**B**) Investigation of cytotoxicity of NK-92 cells against SNU719_AR(−) cells treated with 100 nM DHT for 24 h by LDH release-based cytotoxicity assay. Cytotoxicity of NK-92 cells against SNU719_AR(−) cells treated with EtOH for 24 h was used as a control. (**C**) Investigation of cytotoxicity of NK-92 cells against YCCEL1 cells treated with 100 nM DHT for 24 h by LDH release-based cytotoxicity assay. Cytotoxicity of NK-92 cells against YCCEL1 cells treated with EtOH for 24 h was used as a control. (**D**) Analysis of transcriptional patterns of DHT-regulated NK-cell-activation receptor genes. The transcription of NK-cell-activation receptor genes in NK-92 cells treated with 100 nM DHT for 24 h was investigated by RT-qPCR assay. One-way ANOVA and Tukey’s multiple comparisons test were used for statistical processing. (**E**) Analysis of transcriptional patterns of NK-cell-activating receptor ligand genes regulated by DHT. The transcription of ligand genes of NK-cell-activating receptor in SNU719 cells treated with 100 nM DHT for 24 h was investigated by RT-qPCR assay. One-way ANOVA and Tukey’s multiple comparisons test were used for statistical processing. (**F**) Investigation of cytotoxicity of NK-92 cells against SNU719_pLKO.1 cells treated with 100 nM DHT for 24 h by LDH release-based cytotoxicity assay. Cytotoxicity of NK-92 cells against SNU719_pLKO.1 cells treated with EtOH for 24 h was used as a control. (**G**) Investigation of cytotoxicity of NK-92 cells against SNU719_MICA(−) cells treated with 100 nM DHT for 24 h by LDH release-based cytotoxicity assay. Cytotoxicity of NK-92 cells against SNU719_MICA(−) cells treated with EtOH for 24 h was used as a control. (**H**) Investigation of cytotoxicity of NK-92 cells against SNU719_HLA-A(−) cells treated with 100 nM DHT for 24 h by LDH release-based cytotoxocity assay. Cytotoxicity of NK-92 cells against SNU719_HLA-A(−) cells treated with EtOH for 24 h was used as a control. E (effector cells) and T (target cells) refer to NK-92 cells and SNU719 cells, respectively. The E:T ratio represents the ratio of the number of effector cells to target cells. An E:T ratio of 1:1 corresponds to a combination of 5 × 10^3^ NK-92 cells and 5 × 10^3^ SNU719 cells. We performed a one-way ANOVA test for comparisons involving more than three groups, followed by Tukey’s multiple comparison test if the results were significant. The results were presented using a compact letter display, with both uppercase letters used to denote significant differences.

Therefore, we hypothesized that the killing power of NK cells would be increased by upregulation of ligands of NK-cell receptors activating EBVaGC cells treated with DHT. To test this hypothesis, we investigated the cytotoxicity of NK-92-cells against DHT-treated SNU719 and YCCEL1 cells by lactate dehydrogenase (LDH) release-cytotoxicity assay. SNU719_AR(+) cells were treated with 100 nM DHT and control EtOH for 24 h, and then NK-92 cells were co-cultured with these SNU719_AR(+) cells for 24 h, and the cytotoxicity of NK-92 cells against SNU719_AR(+) cells was measured. NK-92 cytotoxicity was significantly higher in DHT-treated cocultures compared to the control, EtOH-treated cocultures (Figure 3A). Similarly, SNU719_AR(−) cells were treated with 100 nM DHT and control EtOH for 24 h, and then NK-92 cells were co-cultured with these SNU719_AR(−) cells for 24 h to measure the cytotoxicity of NK-92 cells against SNU719_AR(−) cells. NK-92 cytotoxicity was not statistically significant in DHT-treated co-cultures compared to the control, EtOH-treated co-cultures (Figure 3B). YCCEL1 cells were treated with 100 nM DHT and control EtOH for 24 h, and then NK-92 cells were co-cultured with these YCCEL1 cells for 24 h to measure NK-92 cell cytotoxicity against YCCEL1 cells. NK-92 cell cytotoxicity was significantly higher in DHT-treated cultures than in control EtOH-treated cultures (Figure 3C).

The results indicated that DHT enhanced the cytotoxic activity of NK-92 cells against SNU719 and YCCEL1 cells, leading us to hypothesize that this effect was linked to the DHT-induced increase in the interaction between MICA and its corresponding receptors.

Therefore, we analyzed the expression pattern of all NK-cell-activating receptors on NK-92 cells upon DHT treatment (Figure 3D). In details, we investigated changes in transcription of NK-cell-activating receptor genes in NK-92 cells treated with 100 nM DHT for 24 h. Among the nine activated NK-cell receptor genes tested, DHT slightly increased the receptor transcription of *NKp46*, *NTB-4* and *NKG2D*, but this increase was not statistically significant.

Next, the expression pattern of the above ligands of NK-cell-activating receptors was also extensively analyzed. In detail, we investigated changes in the transcription of ligands of NK-cell-activating receptor genes in SNU719 cells treated with 100 nM DHT for 24 h.

**Figure 4:**
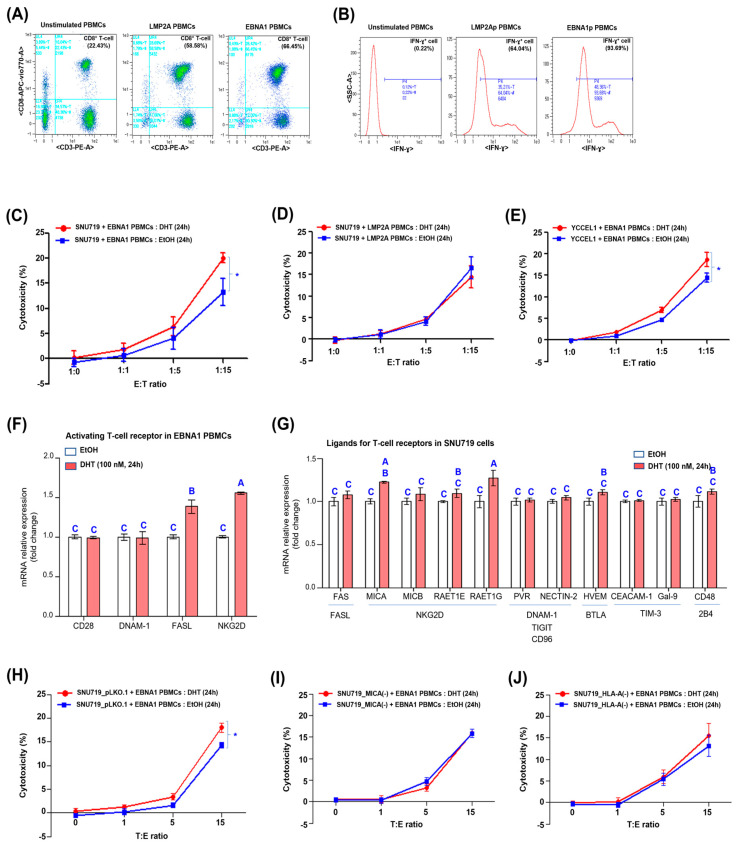
**DHT increases the cytotoxicity of PBMCs.** (**A**) Fresh PBMCs were treated (stimulated) with EBV EBNA1 full peptide (5 g/mL) and LMP2A full peptide (5 g/mL) for 14 days to establish EBNA1 PBMCs and LMP2A PBMCs, respectively. The proportion of CD8^+^ T cells in each of these two PBMCs was investigated by FACs analysis. The bright cyan letters and numbers in the dot plot represent the intensity and percentage of antibody binding to the antigen. Specifically, UL4 indicates the percentage of CD8-positive and CD3-negative cells, LL4 represents the percentage of CD8-negative and CD3-negative cells, UR4 corresponds to the percentage of CD8-positive and CD3-positive cells, and LR4 denotes the percentage of CD8-negative and CD3-positive cells. 1e1 means 1 × 10^1^, which equals 10. 1e2 means 1 × 10^2^, which equals 100. 1e3 means 1 × 10^3^, which equals 1000. (**B**) The proportion of cells secreting INF-γ in each EBNA1 PBMCs and LMP2A PBMCs was examined by FACs analysis. (**C**) Investigation of cytotoxicity of EBNA1 PBMCs against SNU719 cells treated with 100 nM DHT for 24 h by LDH release-based cytotoxicity assay. Cytotoxicity of EBNA1 PBMCs against SNU719 cells treated with EtOH for 24 h was used as a control. (**D**) Investigation of cytotoxicity of LMP2A PBMCs against SNU719 cells treated with 100 nM DHT for 24 h by LDH release-based cytotoxicity assay. Cytotoxicity of LMP2A cells against SNU719 cells treated with EtOH for 24 h was used as a control. (**E**) Investigation of cytotoxicity of EBNA1 PBMCs against YCCEL1 cells treated with 100 nM DHT for 24 h by LDH release-based cytotoxicity assay. Cytotoxicity of EBNA1 PBMCs against YCCEL1 cells treated with EtOH for 24 h was used as a control. (**F**) Analysis of transcriptional patterns of DHT-regulated T-cell-activation receptor genes. The transcription of T-cell-activation receptor genes in EBNA1 PBMCs treated with 100 nM DHT for 24 h was investigated by RT-qPCR assay. One-way ANOVA and Tukey’s multiple comparisons test were used for statistical processing. (**G**) Analysis of transcriptional patterns of T-cell-activating receptor ligand genes regulated by DHT. The transcription of ligand genes of T-cell-activating receptor in SNU719 cells treated with 100 nM DHT for 24 h was investigated by RT-qPCR assay. One-way ANOVA and Tukey’s multiple comparisons test were used for statistical processing. (**H**) Investigation of cytotoxicity of EBNA1 PBMCs against SNU719_pLKO.1 cells treated with 100 nM DHT for 24 h by LDH release-based cytotoxicity assay. Cytotoxicity of EBNA1 PBMCs against SNU719_pLKO.1 cells treated with EtOH for 24 h was used as a control. (**I**) Investigation of cytotoxicity of EBNA1 PBMCs against SNU719_MICA(−) cells treated with 100 nM DHT for 24 h by LDH release-based cytotoxicity assay. Cytotoxicity of EBNA1 PBMCs against SNU719_MICA(−) cells treated with EtOH for 24 h was used as a control. (**J**) Investigation of cytotoxicity of EBNA1 PBMCs against SNU719_HLA-A(−) cells treated with 100 nM DHT for 24 h by LDH release-based cytotoxicity assay. Cytotoxicity of EBNA1 PBMCs against SNU719_HLA-A(−) cells treated with EtOH for 24 h was used as a control. E (effector cells) and T (target cells) refer to PBMCs and SNU719 cells, respectively. The E:T ratio represents the ratio of the number of effector cells to target cells. An E:T ratio of 1:1 corresponds to a combination of 5 × 10^3^ PBMCs and 5 × 10^3^ SNU719 cells. We performed a one-way ANOVA test for comparisons involving more than three groups, followed by Tukey’s multiple comparison test if the results were significant. The results were presented using a compact letter display, with uppercase letters used to denote significant differences. Statistically significant differences identified by the two-tailed Student’s *t*-test are indicated by asterisks (*).

Regarding the ligand genes for NK-cell receptors, DHT statistically significantly increased the transcription of *MICA* (ligand of NKG2D), *RAET1G* (ligand of NKG2D), and *NTB-A* (ligand of NTB-A) among nine ligands (Figure 4E).

The above results suggest that the increased NK-92 cytotoxicity against SNU719 cells by DHT is mainly due to the increased expression of NK-92 cell NKG2D ligands such as MICA and RAET1G. NKG2D is an activation receptor for NK and CD8^+^ T cells, and MICA is a ligand for NKG2D [22]. Previous experiments have shown that DHT increases the transcription of *MICA* and *RAET1G*. Therefore, we hypothesized that DHT-mediated increased NK-cell cytotoxicity is associated with the DHT-mediated upregulation of *MICA*. To test this hypothesis, we knocked down *MICA* using an shRNA lentiviral system to generate SNU719_MICA(−) cells (Supplementary Figure S1C,D). As an internal control, SNU719_HLA-A(−) cells were generated by knockdown of *HLA-A* (Supplementary Figure S1E,F). HLA-A is a ligand for the NK-cell-activating receptor KIR2DS4 and the NK-cell inhibitory receptor KIR3DL2 [22], and is one of the three major types of MHC class I transmembrane proteins [23]. As a negative control, negative control cells were constructed by transfecting SNU719 cells with the shRNA expression vector pLKO.1. These negative control cells were named SNU719_pLKO.1 cells.

The DHT-mediated cytotoxicity of NK cells against SNU719_MICA(−) and SNU719_HLA-A(−) cells was then assessed by LDH release-based cytotoxicity assay. The DHT-mediated cytotoxicity of NK-92 cells against SNU719_pLKO.1, SNU719_MICA(−) and SNU719_HLA-A(−) cells was assessed. As expected, compared to EtOH treatment (control), DHT significantly increased NK-cell cytotoxicity against SNU719_pLKO.1 cells at T:E = 1:15 (Figure 3F). However, compared to the control, DHT significantly decreased NK-cell cytotoxicity against SNU719_MICA(−) cells at T:E = 1:15 (Figure 3G). Interestingly, compared to the control, DHT did not significantly change NK-cell cytotoxicity against SNU719_HLA-A(−) cells at T:E = 1:15 (Figure 3H). Based on these results, it is proposed that the DHT-mediated increase in NK-cell cytotoxicity against SNU719 cells is based on the interaction of NKG2D and MICA.

### 3.4. DHT Increases the Cytotoxicity of PBMCs

The role of T cells is as important as that of NK cells in tumor clearance [24]. We cannot exclude the possibility that DHT may enhance the cytotoxicity of CD8^+^ T cells against SNU719 cells as much as NK cells. To confirm this, PBMCs were isolated from donor blood, and the HLA-A type of these PBMCs was determined by the sequencing of HLA-A exon 2 (Supplementary Figure S3A) and *HLA-A* exon 4 (Supplementary Figure S3B). In addition, EBVaGC cell lines with HLA-A types such as SNU719, YCCEL1, and NCC24 cells were identified.

The PBMCs from donor blood, SNU719 cells and YCCEL1 cells were identified as HLA-A*24:02 type. However, NCC24 cells were identified as HLA-A*11:01:01 type (Supplementary Figure S3). Therefore, SNU719 and YCCEL1 cells were used to investigate the cytotoxicity of the aforementioned PBMCs by LDH release-based cytotoxicity assay.

The PBMCs were then treated with EBBA1 and LMP2A peptides for 14 days. As a result, the PBMCs were stimulated, increasing the proportion of CD 8^+^ T-cells in the PBMCs. Depending on the EBV peptide used, EBNA1 PBMCs and LMP2A PBMCs were constructed. Flow cytometry was used to examine the expression levels of CD8^+^ and CD3^+^ in EBNA1 and LMP2A PBMCs to measure the proportion of CD8^+^ T-cells in the PBMCs (Figure 4A). While unstimulated PBMCs had 22.43% CD8^+^ T-cells, EBNA1 PBMCs had 58.58% CD8^+^ T-cells and LMP2A PBMCs had 66.45% CD8^+^ T-cells. We also measured the amount of IFN-γ+ secreted by EBNA1 and LMP2A PBMCs using intracellular flow cytometry and determined the proportion of IFN-γ+ cells (Figure 4B). While unstimulated PBMCs had 0.22% IFN-γ+ cells, EBNA1 PBMCs had 99.02% IFN-γ+ cells and LMP2A PBMCs had 97.16% IFN-γ+ cells.

The DHT-treated EBNA1 PBMCs showed significantly higher cytotoxicity against SNU719 cells compared to the EtOH-treated control (Figure 4C). However, there was no significant difference in the cytotoxicity of DHT-treated LMP2A PBMCs against SNU719 cells compared to the control (Figure 4D). Additionally, the DHT-treated EBNA1 PBMCs exhibited significantly higher cytotoxicity against YCCEL1 cells than the EtOH-treated control (Figure 4E).

All these results suggested that PBMCs stimulated with EBNA1 peptide exhibited increased cytotoxicity against SNU719 cells by DHT treatment, and that this increased cytotoxicity was due to increased antigen presentation resulting from increased *EBNA1* transcription in SNU719 cells.

In the above results, DHT increased the EBNA1 PBMC killing of SNU719 cells. This prompted us to investigate how DHT regulates the transcript expression of T-cell-activating receptors in the PBMCs. Therefore, we analyzed the changes in transcription patterns of T-cell-activation receptor genes in EBNA1 PBMCs treated with 100 nM DHT for 24 h by RT-qPCR assay. Among the four activated T-cell receptor genes tested, DHT treatment statistically significantly increased the transcript levels of *FASL* and *NKG2D* (Figure 4F).

Next, DHT-induced transcriptional changes in SNU719 cell ligand genes were analyzed using RT-qPCR assay. Among the 11 ligand genes tested, DHT significantly increased the expression of *MICA* and *RAET1G*, both ligands for NKG2D (Figure 4G). These results suggest that the increased cytotoxicity of PBMCs against SNU719 cells by DHT is mainly due to the increased expression of NKG2D on CD8^+^ T-cells and MICA on SNU719 cells. We hypothesized that EBNA1 PBMC cytotoxicity against SNU719 cells requires the interaction of NKG2D and MICA, similar to NK-92 cell cytotoxicity, and tested this hypothesis. The cytotoxicity of EBNA1 PBMCs against SNU719_pLKO.1, SNU719_MICA(−), and SNU719_HLA-A(−) cells was assessed using an LDH release-based cytotoxicity assay. DHT significantly increased the cytotoxicity of EBNA1 PBMCs against SNU719_pLKO.1 cells at T:E = 1:15 compared to EtOH (control) (Figure 4H). However, DHT did not significantly affect the cytotoxicity of EBNA1 PBMCs against SNU719_MICA(−) or SNU719_HLA-A(−) cells at T:E = 1:15 (Figure 4I,J). These results indicate that the DHT-induced increase in EBNA1 PBMCs cytotoxicity against SNU719 cells depends on the interaction between NKG2D and MICA.

### 3.5. Upregulation of EBNA1 and MICA Mediates the DHT-Mediated Cytotoxicity

**Figure 5:**
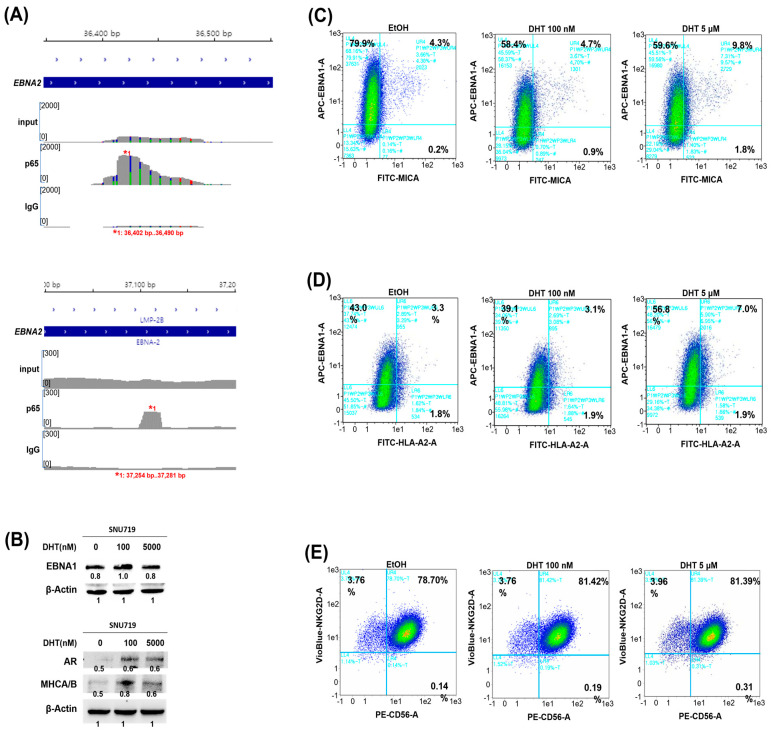
Upregulation of *EBNA1* and *MICA* mediates the DHT-mediated cytotoxicity. (**A**) Investigation of the DHT-regulated p65 enrichment in the EBV EBNA2 exon region. p65 ChIP-seq analysis was performed to identify genomic loci resulting in the p65 enrichment in the genome of SNU719 cells treated with 100 nM DHT for 30 min. Among them, the p65 enrichment at the 36,400–36,500 bp (top) and 37,050–37,150 bp (bottom) loci of the EBV genome was analyzed. (**B**) Investigation of the expression of *EBNA1* in DHT-treated SNU719 cells. SNU719 cells treated with DHT for 48 h were examined for changes in *EBNA1* expression by Western blot assay. *EBNA1* was upregulated, and at the same time, *AR* and *MICA* were also upregulated. (**C**) FACs analysis was performed to investigate the co-expression of *MICA* and *EBNA1*. In SNU719 cells treated with DHT for 48 h, MICA antibody and EBNA1 antibody were reacted without permeabilization, and changes in the expression of *MICA* and *EBNA1* on the cell surface were detected by FACs analysis. The bright cyan letters and numbers in the dot plot represent the intensity and percentage of antibody binding to the antigen. Specifically, UL4 indicates the percentage of EBNA1-positive and MICA-negative cells, LL4 represents the percentage of EBNA1-negative and MICA-negative cells, UR4 corresponds to the percentage of EBNA1-positive and MICA-positive cells, and LR4 denotes the percentage of EBNA1-negative and MICA-positive cells. (**D**) FACs analysis was performed to investigate the co-expression of *HLA-A* and *EBNA1*. HLA-A and EBNA1 antibodies were reacted with SNU719 cells treated with DHT for 48 h without permeabilization, and changes in the expression of *HLA-A* and *EBNA1* on the cell surface were determined by FACs analysis. The bright cyan letters and numbers in the dot plot represent the intensity and percentage of antibody binding to the antigen. Specifically, UL4 indicates the percentage of EBNA1-positive and HLA-A2-negative cells, LL4 represents the percentage of EBNA1-negative and HLA-A2-negative cells, UR4 corresponds to the percentage of EBNA1-positive and HLA-A2-positive cells, and LR4 denotes the percentage of EBNA1-negative and HLA-A2-positive cells. (**E**) FACs analysis was performed to investigate the co-expression of *CD56* and *NKG2D*. CD56 and NKG2D antibodies were reacted with NK-92 cells treated with DHT for 48 h without permeabilization, and changes in the expression of *CD56* and *NKG2D* on the cell surface were determined by FACs analysis. The bright cyan letters and numbers in the dot plot represent the intensity and percentage of antibody binding to the antigen. Specifically, UL4 indicates the percentage of NKG2D-positive and CD56-negative cells, LL4 represents the percentage of NKG2D-negative and CD56-negative cells, UR4 corresponds to the percentage of NKG2D-positive and CD56-positive cells, and LR4 denotes the percentage of NKG2D-negative and CD56-positive cells. 1e1 means 1 × 10^1^, which equals 10. 1e2 means 1 × 10^2^, which equals 100. 1e3 means 1 × 10^3^, which equals 1000.

The putative molecular mechanism for the increased cytotoxicity of NK-92 cells and EBNA1 PBMCs against DHT-treated SNU719 cells is as follows. It was proposed that *EBNA1* and *MICA* were upregulated by the increased transcriptional activator NF-κB in DHT-treated SNU719 cells and that they were more easily recognized by the NKG2D receptor of NK cells and EBNA1, resulting in increased cytotoxicity. Therefore, we examined all the loci enriched by the p65 protein on the EBV genome in the p65 ChIP-seq data, using SNU719 cells treated with 100 nM DHT for 30 min (Supplementary Figure S4). Interestingly, the p65 protein was enriched at approximately nine loci, with the strongest and second-strongest enrichment at the 36,402–36,490 bp locus and the 60,482–60,500 bp locus of the EBV genome, respectively (Figure 5A and Supplementary Figure S4E). Among the above two loci, the 36,402–36,490 bp locus of the EBV genome was relatively close to Wp, which is located near 30,000 bp [25]. Wp is primarily involved in regulating the early expression of EBNA2 and other EBNA proteins, while Cp is involved in *EBNA1* expression in some circumstances [24,25].

Next, we investigated the upregulation of EBNA1 in response to DHT by Western blot (Figure 5B). In SNU719 cells treated with 100 nM DHT, *EBNA1*, *MHCA/B*, and *AR* expression increased compared to the control. However, at 5000 nM DHT, there was no increase in *EBNA1*, *AR*, or *MHCA/B* expression.

We also utilized FACS to investigate whether *MICA* upregulation in DHT-treated SNU719 cells was associated with increased EBNA1 expression (Figure 5C). As the concentration of DHT increased, the number of MICA^+^ SNU719 cells rose, while the number of EBNA1^+^ cells decreased. However, the population of MICA^+^/EBNA1^+^ cells showed a slight increase at 100 nM and more than doubled significantly at 5000 nM. Additionally, FACS analysis was used to assess HLA-A and EBNA1 expression in SNU719 cells treated with DHT (Figure 5D). The number of HLA-A^+^ cells exhibited minimal change with increasing DHT concentration, whereas EBNA1^+^ cells showed concentration-independent fluctuations. The HLA-A^+^/EBNA1^+^ cell population slightly decreased at 100 nM but significantly increased, more than doubling, at 5000 nM. Lastly, we used FACS to examine CD56 and NKG2D expression on NK-92 cells treated with DHT (Figure 5E). The number of CD56^+^ cells increased with rising DHT concentrations, while NKG2D^+^ cells showed a slight increase at 5000 nM. The CD56^+^/NKG2D^+^ cell population also increased marginally with increasing DHT concentrations. These findings suggest that the enhanced cytotoxicity of NK-92 cells and PBMCs against SNU719 cells is due to the DHT-induced upregulation of *EBNA1* and *MICA* in SNU719 cells. The upregulation of *NKG2D* by DHT is not considered a contributing factor to the increased cytotoxicity of NK-92 cells. Specifically, *MICA* upregulation by DHT enhances cytotoxicity by promoting NKG2D receptor recognition on NK cells. Similarly, DHT-induced *EBNA1* upregulation enhances CD8^+^ T-cell recognition via MHC-I antigen presentation, leading to increased cytotoxicity.

### 3.6. DHT Does Not Regulate Cytokine Expression

**Figure 6:**
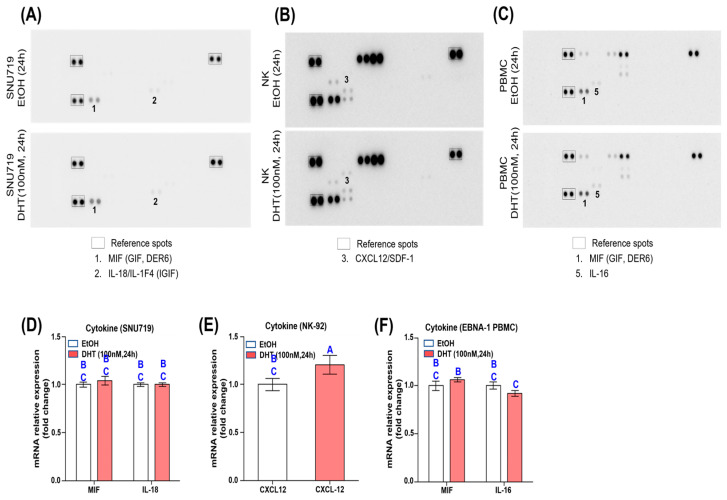
**DHT does not regulate cytokine expression.** (**A**) Cytokine array analysis to identify cytokines secreted from SNU719 cells treated with 100 nM DHT for 24 h. (**B**) Cytokine array analysis to identify cytokines secreted from NK-92 cells treated with 100 nM DHT for 24 h. (**C**) Cytokine array analysis to identify cytokines secreted from EBNA1 PBMCs treated with 100 nM DHT for 24 h. (**D**) Analysis of transcriptional changes of *MIF1* and *IL-18* genes in SNU719 cells treated with 100 nM DHT for 24 h by RT-qPCR assay. (**E**) Analysis of transcriptional changes of *CXCL12* gene in NK-92 cells treated with 100 nM DHT for 24 h by RT-qPCR assay. (**F**) Analysis of transcriptional changes of *MIF1* and *IL-16* genes in EBNA1 PBMCs treated with 100 nM DHT for 24 h by RT-qPCR assay. We performed a one-way ANOVA test for comparisons involving more than three groups, followed by Tukey’s multiple comparison test if the results were significant. The results were presented using a compact letter display, with both uppercase letters used to denote significant differences.

Previous studies have shown that DHT treatment inhibits the expression of the proinflammatory cytokine IL-6 and increases the expression of IL-10 [26,27]. Therefore, we investigated changes in cytokine expression in DHT-treated SNU719 cells, NK-92 cells, and EBNA1 PBMCs using a cytokine array kit that detects 36 cytokines simultaneously. First, SNU719 cells treated with 100 nM DHT for 24 h showed a slight increase in MIF and IL-18/IL-1F4 (Figure 6A). Second, CXCL12/SDF-1 slightly decreased in NK cells treated with 100 nM DHT for 24 h (Figure 6B). Third, EBNA1 PBMCs treated with 100 nM DHT for 24 h showed a slight increase in MIF and IL-16 (Figure 6C). Overall, DHT treatment slightly increased or decreased some cytokines. To validate these changes, we performed RT-qPCR assays. Changes in *MIF* and *IL-18* transcripts in SNU719 cells treated with 100 nM DHT for 24 h were not statistically significant (Figure 6D). Additionally, *CXCL12* transcripts in NK-92 cells treated with 100 nM DHT for 24 h were significantly increased compared to the control (Figure 6E). Finally, changes in *MIF* and *IL-16* transcripts in EBNA1 PBMCs treated with 100 nM DHT for 24 h were not statistically significant (Figure 6F). These results indicate that, contrary to our expectations and previous studies, DHT treatment did not strongly increase cytokine expression, suggesting that cytokines are not deeply involved in the DHT-induced increase in cytotoxicity of cellular immune cells.

## 4. Discussion

The role of male hormones, particularly dihydrotestosterone (DHT), in the progression and treatment of Epstein–Barr virus-associated gastric carcinoma (EBVaGC) has been underexplored compared to other cancers. Our study provides new insights into how DHT modulates the immune response against EBVaGC, highlighting its potential therapeutic implications. We observed that DHT significantly increases androgen receptor (*AR*) expression in EBVaGC cells. The DHT-AR complex activates various signaling pathways, including the upregulation of NF-κB, which is crucial for immune response modulation. This upregulation was associated with an increased expression of MICA protein, a critical ligand for the NKG2D receptor on natural killer (NK) and T cells. Increased MICA levels enhance interaction with the NKG2D receptor, boosting NK- and T-cell cytotoxicity against SNU719 cells—an EBVaGC cell line. This immune activation occurs without a concomitant increase in proinflammatory cytokine gene expression, suggesting a targeted and potentially less-inflammatory immune response.

Previous studies have shown that DHT, along with DHEA and E2, reduces IL-6 expression in human bone-marrow cells and that testosterone replacement in androgen-deficient males decreases IL-10 expression [26,27]. These findings suggest that male hormones modulate immune responses by regulating inflammatory cytokines in normal cells, but this modulation does not seem effective in cancer cells. In EBVaGC, no significant changes in cytokine expression were noted after 24 h treatment with 100 nM DHT, despite the sufficient induction of gene expression via NF-κB. This suggests that DHT-AR signaling may contribute more to direct NK- and T-cell activation rather than relying on NF-κB to upregulate inflammatory cytokine genes.

NKG2D, expressed on T and NK cells, transmits activation signals by recognizing NKG2D ligands (NKG2DLs) on tumor cells. Among these ligands, MICA and MICB are well characterized, with MICA more abundantly expressed in tumor cells [28,29]. Our analysis revealed that DHT treatment upregulates *MICA* and *RAET1G* expression in EBVaGC, with a more substantial increase in *MICA*. The upregulation of *MICA* by DHT counteracts the reduction caused by MICA/B shedding, enhancing NK-cell recognition and suppressing immune escape [30,31].

We generated Epstein–Barr virus (EBV)-specific peripheral blood mononuclear cells (PBMCs) targeting EBNA-1 (EBNA1 PBMCs) and LMP2A (LMP2A PBMCs), proteins expressed during the EBV latent phase in EBVaGC [32]. Notably, DHT treatment only enhanced the cytotoxicity of EBNA1 PBMCs. Given that EBNA-1 is expressed in nearly all EBV latent phases, EBNA-1 peptides could serve as target antigens in most EBV-associated malignancies. Although EBNA-1 interferes with HLA-A presentation [28], DHT treatment did not upregulate *HLA-A* expression in SNU719 cells. However, HLA-A knockdown slightly reduced the DHT-mediated cytotoxicity of EBNA1 PBMCs, suggesting that DHT may partially restore the HLA-A presentation of EBNA-1.

Adoptive cell therapy (ACT) with EBV-specific T cells has been used for over 25 years to treat EBV-associated malignancies [33], but its application to solid tumors like EBVaGC is challenging due to the immunosuppressive tumor microenvironment (TME) [34]. Our study confirms that DHT upregulates *MICA* in SNU719 cells and *NKG2D* in EBNA1 PBMCs and NK-92 cells, enhancing the interaction between MICA and NKG2D and boosting cytotoxicity against SNU719 cells. Combining DHT with immune checkpoint inhibitors, which block PD-1 and PD-L1 [35], could further enhance this cytotoxicity.

**Figure 7:**
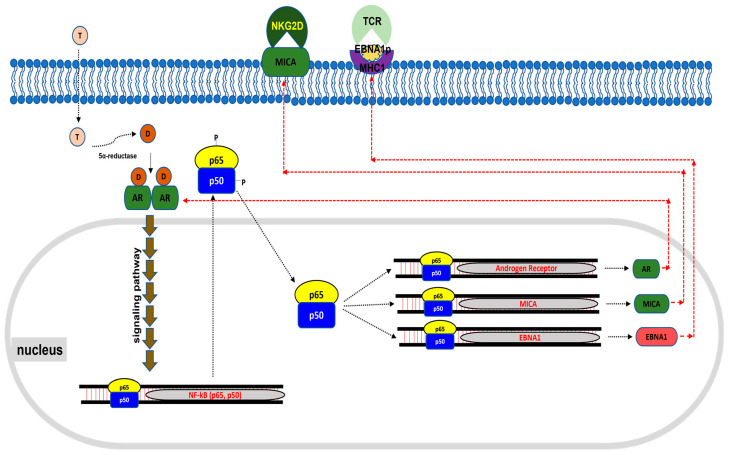
**Schematic illustrating the molecular mechanism by which DHT increases the cellular response of cellular immunity.** DHT enters the cell, binds to the AR, and upregulates NF-κB through signaling. This upregulated NF-κB is phosphorylated in the cytoplasm and enters the nucleus where it acts as a transcriptional activator. This transcriptional activator promotes the transcription of *AR*, *MICA* and *EBNA1*. The resulting AR is continuously bound to DHT to maintain signal transduction. The resulting MICA translocates to the cell membrane and acts as a ligand for the activating receptor NKG2D on NK cells and CD8^+^ T cells. The resulting EBNA1 is then used as an antigenic peptide for MHC-1, which reacts with the main receptor (TCR) of CD8^+^ T cells.

We were unable to successfully conduct planned animal experiments due to challenges with tumorigenesis in humanized mice. Future studies should address these issues. Our findings suggest that the DHT-AR complex activates key signaling pathways that enhance MICA expression and EBNA1 presentation, boosting the immune response against EBVaGC (Figure 7). Further clinical validation and investigations into the mechanisms underlying the MHC class I presentation of EBNA1 peptides regulated by DHT-AR signaling are warranted. This study is significant as it is the first to demonstrate that male hormones can enhance antitumor immunity against EBVaGC, suggesting new therapeutic strategies. Overall, this study underscores the potential of leveraging DHT to modulate immune responses and improve treatment outcomes for EBVaGC patients.

## 5. Conclusions

Our study demonstrates that dihydrotestosterone (DHT) enhances the immune response against EBV-associated gastric carcinoma by upregulating MICA expression and activating NK and T cells, without inducing proinflammatory cytokines. Targeting androgen signaling may offer novel therapeutic strategies for improving anti-tumor immunity in EBVaGC.

## Data Availability

All relevant data are within the paper and its Supporting Information files.

## Supplementary Materials

The following supporting information can be downloaded at: https://www.mdpi.com/article/10.3390/cancers16183219/s1, Figure S1: Construction of SNU719_AR(−), SNU719_MICA(−), and SNU719_HLA-A(−) cells. (A,B) SNU719_AR(−) cells were generated using the CRISPR/Cas9 system. *AR* transcript and AR protein expression in SNU719_AR(−) cells were analyzed by RT-qPCR (A) and western blotting (B). (C,D) SNU719_MICA(−) cells were generated using the shRNA lentiviral system. *MICA* transcript and MICA protein expression in SNU719_MICA(−) cells were analyzed by RT-qPCR (C) and western blotting (D). (E,F) SNU719_HLA-A(−) cells were generated using the shRNA lentiviral system. *HLA-A* transcript and HLA-A protein expression in SNU719_HLA-A(−) cells were analyzed by RT-qPCR (E) and western blotting (F); Figure S2: p65 enrichment for RAET1G upregulation. ChIP-seq analysis was conducted to identify p65 enrichment sites in the genome of SNU719 cells treated with 100 nM DHT for 30 min. The analysis focused on the enrichment of the p65 protein at the *RAET1G* promoter locus, located at q23.2 of chromosome 6; Figure S3: Identification of HLA-A type in EBVaGC cells. (A) Comparison of *HLA-A* exon 2 sequences. The *HLA-A* 24:02 exon 2 sequence was obtained from NCBI GenBank (accession number: LT883521.1). DNA regions corresponding to *HLA-A* exon 2 from EBVaGC cells and PBMCs were amplified and analyzed using Sanger sequencing. Sequence alignment of exon 2 was performed using the MultAlin interface (multalin.toulouse.inra.fr/multalin/) accessed on 1 October 2023. (B) Comparison of *HLA-A* exon 4 sequences. The *HLA-A* 24:02 exon 4 sequence was obtained from NCBI GenBank (accession number: LT883521.1) accessed on 1 September 2023. DNA regions corresponding to *HLA-A* exon 4 from EBVaGC cells and PBMCs were amplified and analyzed using Sanger sequencing. Sequence alignment of exon 4 was performed using the MultAlin interface (multalin.toulouse.inra.fr/multalin/) accessed on 1 October 2023; Figure S4: DHT-mediated p65 enrichment on the EBV genome. A ChIP-seq assay was conducted to identify genomic loci in the EBV genome of SNU719 cells treated with 100 nM DHT for 30 min, which resulted in p65 enrichment. (A) p65 protein enrichment on the *EBNA2* exon at 36,402-36,490 bp of the EBV genome. (B) p65 protein enrichment on the *EBNA2* exon at 37,254–37,281 bp of the EBV genome. (C) p65 protein enrichment on the *BRFR3* exon at 49,654–49,673 bp of the EBV genome. (D) p65 protein enrichment on the *BLPF1* exon at 54,549–54,814 bp of the EBV genome. (E) p65 protein enrichment on the *BPLF1* promoter at 60,482–60,500 bp of the EBV genome. (F) p65 protein enrichment on the *BSLF2* exon at 71,705–71,732 bp of the EBV genome. (G) p65 protein enrichment on the *BZLF1* exon at 90,481–90,506 bp of the EBV genome. (H) p65 protein enrichment on the *BRRF2* 3′UTR at 95,795–95,945 bp of the EBV genome. (I) p65 protein enrichment on the *BALF2* exon at 162,605–162,622 bp of the EBV genome.
