# Supplementary material for: Dihydrotestosterone Enhances MICA-Mediated Immune Responses to Epstein–Barr Virus-Associated Gastric Carcinoma
Source: Cancers (Basel). 2024 Sep 21;16(18):3219. doi: 10.3390/cancers16183219 (PMC11429822; doi:10.3390/cancers16183219)
Supplement: Supplementary file 1 [file cancers-16-03219-s001.zip › cancers-3194740-supplementary.pdf]

Supporting Information Figure Legends

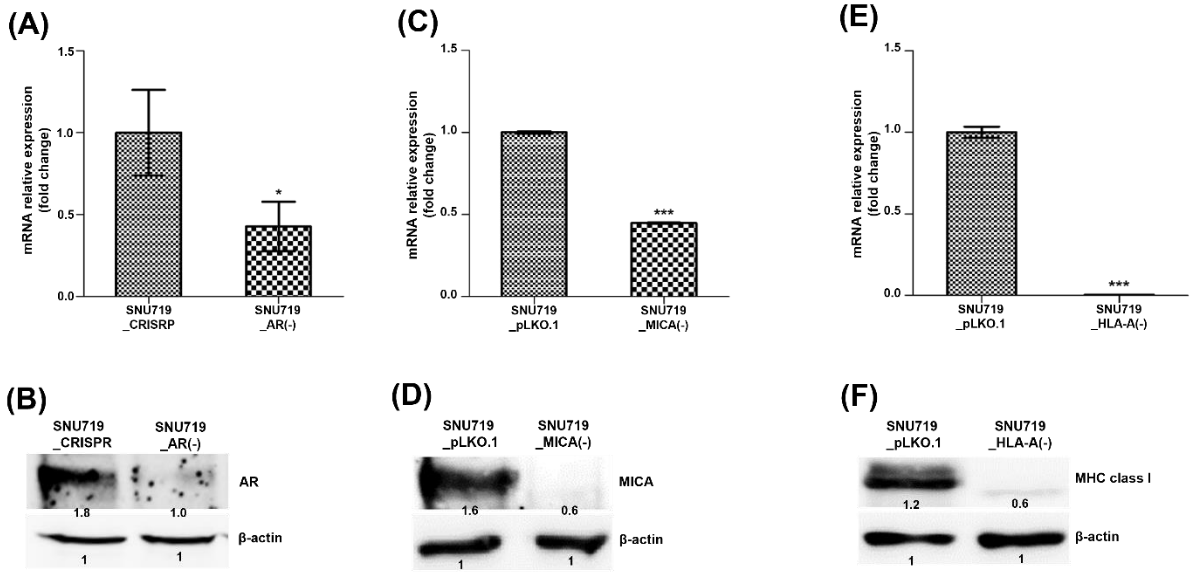

Supplementary Fig. 1 Construction of SNU719_AR(−), SNU719_MICA(−) and SNU719_HLA-A(−) cells.

(A-B) SNU710_AR(−) cells were constructed using the CRISPR/Cas9 system. The expression of the *AR* transcript and AR protein in SNU710_AR(−) cells was investigated by RT-qPCR (A) and western blotting (B).

(C-D) SNU710_MICA(−) cells were constructed using the shRNA lentiviral system. The expression of the *MICA* transcript and MICA protein in SNU710_MICA(−) cells was investigated by RT-qPCR (C) and western blotting (D).

(E-F) SNU710_HLA-A(−) cells were constructed using the shRNA lentiviral system. The expression of the *HLA-A* transcript and HLA-A protein in SNU710_HLA-A(−) cells was investigated by RT-qPCR (E) and western blotting (F).

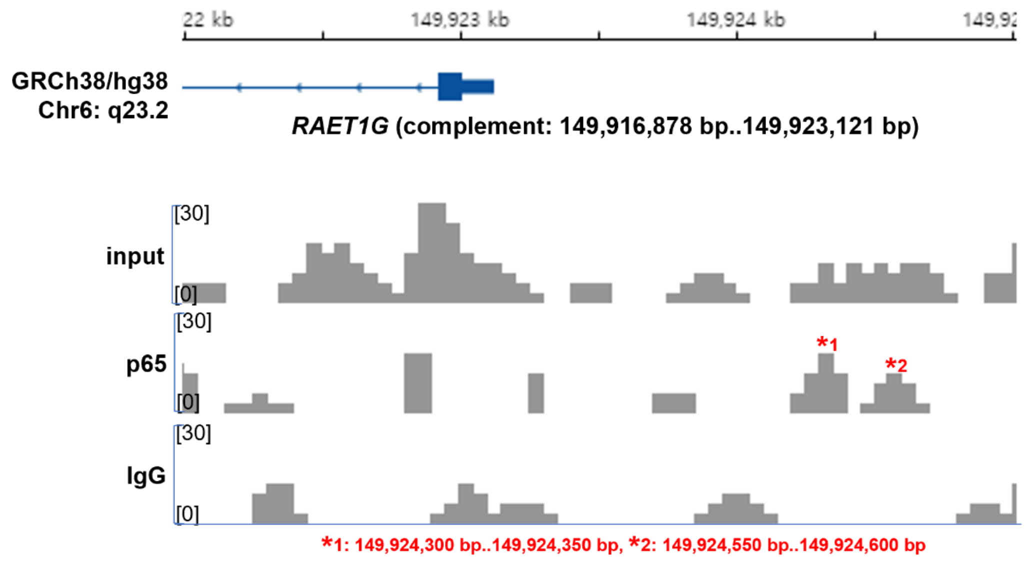

Supplementary Fig. 2 The p65 enrichment for the *RAET1G* upregulation. ChIP-seq analysis was performed to assess the p65 enrichment sites in the genome of SNU719 cells treated with 100 nM DHT for 30 min. Assessment of the p65 protein that enriches the *RAET1G* promoter locus located at q23.2 of chromosome 6.

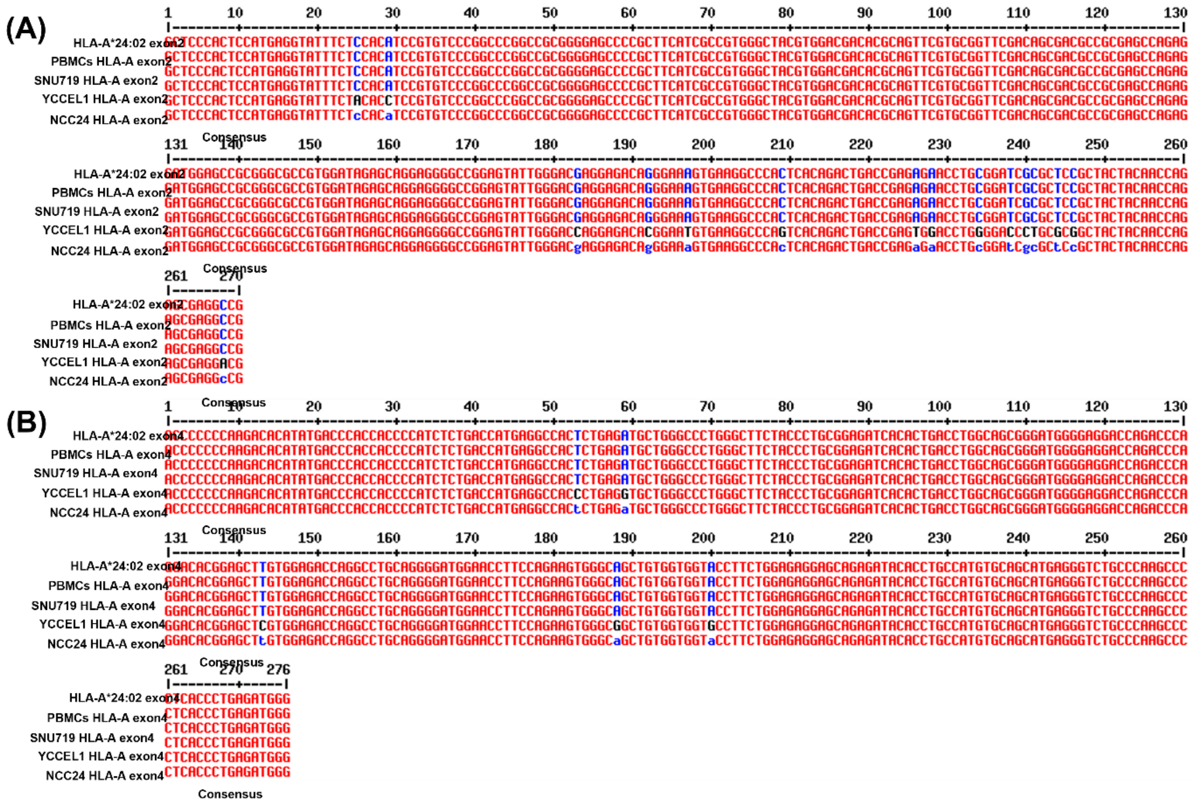

Supplementary Fig. 3 Identification of HLA-A type of EBVaGC cells.

(A) Comparison of HLA-A exon 2 sequences. The HLA-A*24:02 exon sequence was obtained from NCBI GenBank (LT883521.1). DNA regions of HLA-A exon 2 of EBVaGC cells and PBMCs were amplified and subjected to Sanger sequencing. Alignment of exon 2 sequences was performed using the MultAlin interface (multalin.toulouse.inra.fr/multalin/).

(B) Comparison of *HLA-A* exon4 sequences. The HLA-A*24:02 exon sequence was obtained from NCBI GenBank (LT883521.1). DNA regions of *HLA-A* exon 4 of EBVaGC cells and PBMCs were amplified and subjected to Sanger sequencing. Alignment of exon 4 sequences was performed using the MultAlin interface (multalin.toulouse.inra.fr/multalin/).

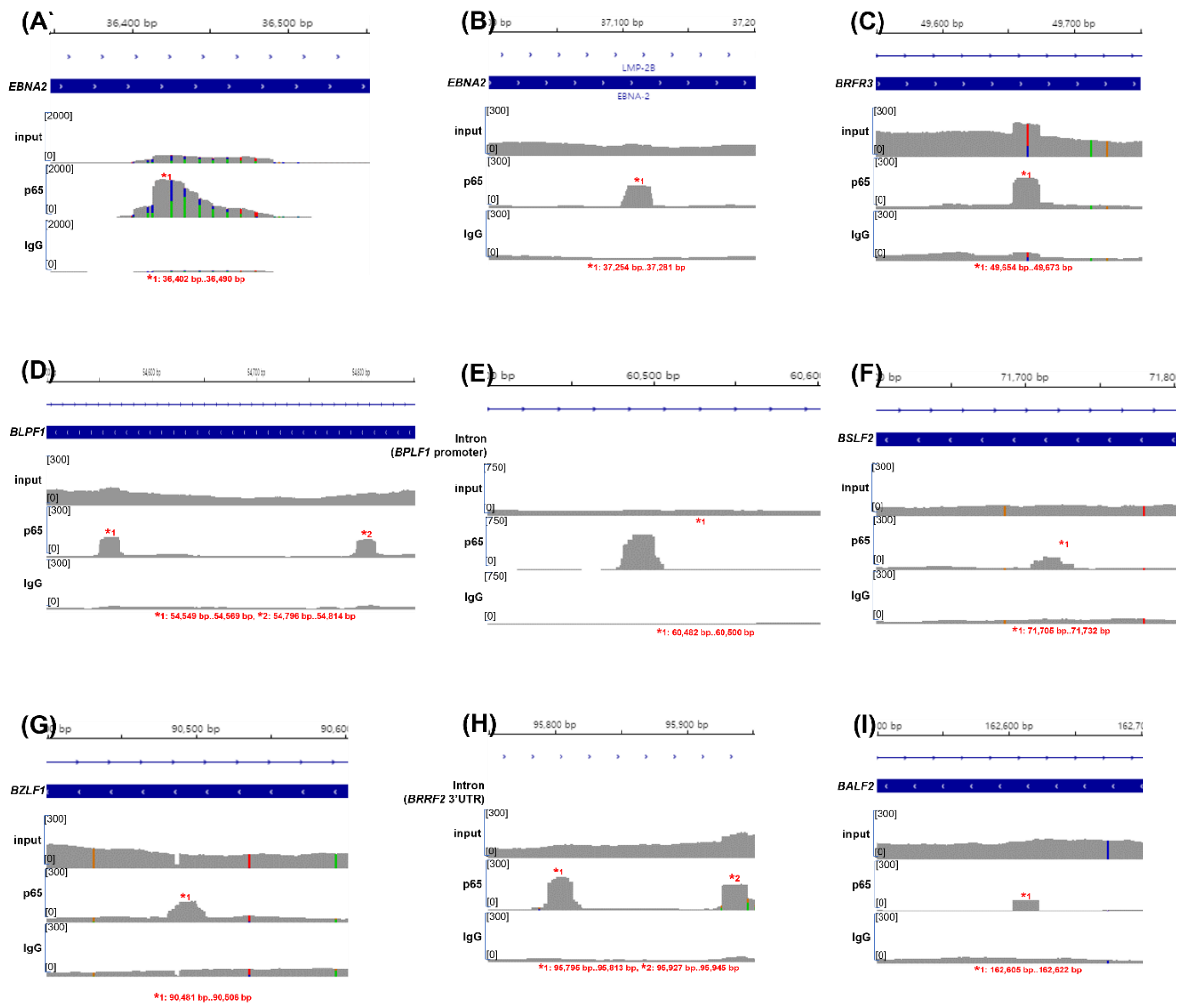

Supplementary Fig. 4 The DHT-mediated p65 enrichment on EBV genome. A ChIP-seq assay was performed to identify the genomic loci in the EBV genome of SNU719 cells treated with 100 nM DHT for 30 min that resulted in the p65 enrichment.

(A) p65 protein enriched on *EBNA2* exon bearing 36,402-36,490 bp of the EBV genome.

(B) p65 protein enriched on *EBNA2* exon bearing 37,254-37,281 bp of the EBV genome.

(C) p65 protein enriched on *BRFR3* exon bearing 49,654-49,673 bp of the EBV genome.

(D) p65 protein enriched on *BLPF1* exon bearing 54,549-54,814 bp of the EBV genome.

(E) p65 protein enriched on *BPLF1* promoter bearing 60,482-60,500 bp of the EBV genome.

(F) p65 protein enriched on *BSLF2* exon bearing 71,705-71,732 bp of the EBV genome.

(G) p65 protein enriched on *BZLF1* exon bearing 90,481-90,506 bp of the EBV genome.

(H) p65 protein enriched on *BRRF2* 3'UTR bearing 95,795-95,945 bp of the EBV genome.

(I) p65 protein enriched on *BALF2* exon bearing 162,605-162,622 bp of the EBV genome.
